# An Investigation of PPy@1T/2H MoS_2_ Composites with Durable Photothermal-Promoted Effect in Photo-Fenton Degradation of Methylene Blue and in Water Evaporation

**DOI:** 10.3390/polym15193900

**Published:** 2023-09-27

**Authors:** Yanhua Lei, Da Huo, Hui Liu, Sha Cheng, Mengchao Ding, Bochen Jiang, Fei Zhang, Yuliang Zhang, Guanhui Gao

**Affiliations:** 1 Institute of Marine Materials Science and Engineering, Shanghai Maritime University, Shanghai 201306, China; huoda18852959827@163.com (D.H.); liang.zai.la@outlook.com (H.L.); dingmengchao111@163.com (M.D.); bochenjiang@hotmail.com (B.J.); ylzhang@shmtu.edu.cn (Y.Z.); 2 Qingdao Product Quality Testing Research Institute, Qingdao 266061, China; xigua5200@163.com; 3 Material Science and Nano engineering Department, Rice University, Houston, TX 77005, USA; gg13@rice.edu

**Keywords:** advanced oxidation processes, photo-Fenton agent, 1T-2H MoS_2_, photothermal water evaporation, photocatalyst

## Abstract

MoS_2_ has garnered considerable attention as an exceptional co-catalyst that is capable of significantly enhancing the efficiency of H_2_O_2_ decomposition in advanced oxidation processes (AOPs). This improvement allows for a reduction in the required amounts of H_2_O_2_ and Fe^2+^. In this study, we investigated the cyclic durability of photo-Fenton catalysts, focusing on the degradation of pollutants through the introduction of PPy into heterogeneous 1T-2H MoS_2_ units. The resulting photothermal-Fenton catalysts, comprising non-ferrous Fenton catalysts, demonstrated excellent degradation performance for simulated pollutants. In comparison with 1T-2H MoS_2_, the PPy@1T-2H MoS_2_ composite exhibited remarkable stability and photothermal enhancement in the photo-Fenton degradation of methylene blue (MB) under visible light irradiation. The photo-Fenton reaction efficiently degraded contaminants, achieving 99% removal within 5 min and 99.8% removal within 30 min. Moreover, the co-catalyst complex displayed enhanced cyclic stability during the photo-Fenton reaction, with a contaminant removal efficiency of 92%, even after the 13th cyclic test. The combined effects of PPy and 1T-2H MoS_2_ demonstrated improved efficiency in both photocatalytic and photo-Fenton catalytic reactions. Furthermore, PPy@1T-2H MoS_2_ exhibited outstanding performance in the photothermal evaporation of water, achieving an efficiency of 86.3% under one solar irradiation.

## 1. Introduction

The scarcity of freshwater resources poses a major challenge to sustainable development today. The misuse of water resources and the escalating water pollution crisis have further exacerbated this scarcity. In addressing the issue of difficult-to-degrade organic wastewater, advanced oxidation processes (AOPs) have emerged as a prominent area of research [1,2,3]. These processes generate powerful oxidizing agents, including ∙SO_4_^2−^ and ∙OH, which can indiscriminately target organic pollutants, ultimately transforming them into CO_2_, H_2_O_2_, and inorganic ions. This capability aligns with the demands of industrial technological advancement and the pursuit of a low-carbon economy [3].

Among the various AOPs, the Fenton process has gained significant popularity due to its effectiveness, simplicity, and cost efficiency. In the conventional Fenton reaction, hydroxyl radicals (∙OH) are generated from the combination of ferrous ions (Fe^2+^) and hydrogen peroxide (H_2_O_2_), which have a redox potential of 2.80 V. However, the efficiency of H_2_O_2_ decomposition is relatively low, which limits the progress of the Fenton reaction. As a result, a larger quantity of H_2_O_2_ and Fe^2+^ is required to attain a sufficient concentration of ∙OH, resulting in the production of substantial amounts of iron sludge. Furthermore, the Fenton reaction is constrained by a narrow pH range, making it challenging to recover the Fe^2+^ reagents following the reaction [4,5].

To overcome the limitations, researchers have explored modified Fenton processes, such as photo-Fenton [6,7] and electro-Fenton [8,9,10]. These approaches aim to enhance the degradation efficiency and address the drawbacks of traditional Fenton reactions. In recent studies, energy sources such as UV and visible light were incorporated into the Fenton system to improve the generation of hydroxyl radicals (·OH) and facilitate Fe^2+^ recovery [11]. Various catalysts and co-catalysts were investigated in photo-Fenton processes, including TiO_2_ [12], CdS [13], Fe_2_O_3_ [14], ZnO [15], WO_3_ [16], g-C_3_N_4_ [17,18], and BiVO_4_ [19,20]. These catalysts have shown significant advancements in promoting the decomposition of H_2_O_2_. However, many of these catalysts face challenges, such as toxicity, the use of expensive reagents, complex preparation processes, low ·OH generation yield, or low recovery efficiency. For instance, although TiO_2_ is an inexpensive photocatalyst with excellent photochemical properties, it has limitations, such as a wide band gap; susceptible recombination; and practical issues, like agglomeration [15]. Therefore, the development of highly active and cost-effective Fenton catalysts remains a challenging task.

In the field of semiconductor photocatalysis, there is currently a growing interest in exploring monolayer or few-layer two-dimensional transition metal compounds, particularly MoS_2_. These materials have garnered significant attention due to their favorable bandgap, abundant active sites, and large specific surface area. Two-dimensional transition metal MoS_2_ has been extensively studied, and MoS_2_ nanomaterials have demonstrated high mobility of photogenerated charge carriers. However, their photocatalytic activity is limited by the fact that the reactive sites are primarily located at the edges of the nanosheets [21,22,23]. To address this challenge, numerous researchers have focused on synthesizing MoS_2_ nanomaterials with a layered structure to enhance their photocatalytic performance [24].

The remarkable properties of MoS_2_ have spurred an increase in studies on its application in the Fenton reaction [25,26,27]. As a co-catalyst, 2D-MoS_2_ not only enhances the degradation performance of pollutants through AOPs in a short timeframe but also exhibits cyclic durability [28]. However, the low electrical conductivity of MoS_2_ nanosheet interfaces poses a limitation to their practical application. To overcome this challenge, researchers have developed composite or heterojunction structures to facilitate the use of MoS_2_ and improve its conductivity [29]. When pure MoS_2_ is used as a photocatalyst, the photogenerated electrons and holes tend to recombine due to its high exciton binding energy, resulting in low utilization of these charge carriers [30]. To address this issue, researchers constructed composite or heterojunction structures to effectively separate the photogenerated electrons and holes in MoS_2_ catalysis, thereby improving their lifetimes [31]. For instance, Tan et al. introduced oxygen-doped MoS_2_ nanosheets (MoS_2_-xOx) as co-catalysts in the Fenton reaction to degrade antibiotic contaminants in water [32]. With the addition of Fe^2+^ and H_2_O_2_, an excellent degradation efficiency of 80% was achieved within 30 min.

In the development of photocatalysis, 2H MoS_2_ has attracted the attention of countless researchers because of its superior light absorption ability and suitable bandgap, but its semiconductor nature limits its electron mobility efficiency, whereas 1T MoS_2_ as a metal phase possesses high conductivity and good electron mobility, which enables the photogenerated electrons produced by light energy excitation to quickly escape from photogenerated holes in the photocatalytic process, thus inhibiting the combination of the two and improving the utilization efficiency of light energy [33]. Research on its use in the Fenton reaction has also gradually increased. However, the stability of 1T-phase MoS_2_ with its metastable structure remains a challenge. The reliance on Fenton reagents and the potential for iron sludge formation remained a concern, leading to the possibility of secondary pollution. The stability of 1T-phase MoS_2_ with its metastable structure can be reduced to 2H-phase MoS_2_ [34,35], even when exposed to air for a short period. The 1T-2H phase of molybdenum disulfide can effectively combine the advantages of the 2H and 1T phases, which effectively promotes the separation of photogenerated electrons and voids and enhances the catalytic activity of the materials. Our preliminary work revealed that 1T-2H MoS2 exhibits good catalytic effects in the photo-Fenton reaction. Nonetheless, the stability of 1T-2H MoS_2_ has been a major issue [36], hindering its further application in photo-catalysts and photo-Fenton reactions.

To improve the stability of 1T-2H MoS_2_ and reduce the recombination of photogenerated electrons and holes, we considered the incorporation of polypyrrole (PPy). In addition, we explored the possibility of designing and synthesizing Fenton catalysts with high photothermal conversion efficiencies to enhance photo-Fenton degradation by increasing the solution temperature [37,38].

Herein, we discuss the development of cyclic durable photothermal-Fenton catalysts by introducing PPy into heterogeneous 1T-2H MoS2 units. The resulting non-ferrous Fenton catalysts exhibited excellent degradation performance for simulated pollutants. The PPy@1T-2H MoS_2_ composites achieved a degradation efficiency of over 92% after 13 cycles, significantly reducing the need for Fe^2+^ and H_2_O_2_ addition. Furthermore, the composites demonstrated excellent photothermal conversion in solar vapor generation, with an efficiency of 86.3% under one solar irradiation. This suggests that the composites can simultaneously enhance the photo-Fenton reaction and facilitate water evaporation, presenting great potential for market application in water treatment.

## 2. Experimental

### 2.1. Chemical Sample Reagents

Thiourea (CH_4_N_2_S, AR, 99 wt%), sodium molybdate dihydrate (Na_2_MoO_4_·2H_2_O, 99 wt%), hydrochloric acid (HCl, 36–38 wt%), propionic acid (C_3_H_6_O_2_, AR, 99.5 wt%), polyvinyl alcohol (PVA), and methylene blue (MB) were all obtained from Alladdin Chemical Reagent Co. (Shanghai, China). Polyvinylidene fluoride (PVDF) was obtained from Shandong West Asia Chemical Industry Co. (Linyi, China). All the reagents and solvents with purity in analytical grade were used directly without any further purification.

### 2.2. Preparation of 1T-2H MoS_2_ and PPy@1T-2H MoS_2_

The 1T-2H MoS_2_ nanomaterials were prepared via the hydrothermal method from precursors composed of sodium molybdate dihydrate (Na_2_MoO_4_·2H_2_O), thiourea (CS(NH_2_)_2_), and propionic acid (C_3_H_6_O_2_). First, Na_2_MoO_4_·2H_2_O (1 mmol), CS(NH_2_)_2_ (3 mmol), and C_3_H_6_O_2_ (8 mL) were placed in 35 mL of deionized water and sonicated until the solution was homogeneous. The mixed solution was then transferred to a stainless-steel reactor with a polytetrafluoroethylene liner and placed in an oven at 180 °C for 4 h. After the reactor had cooled naturally, the precipitates were extracted, filtered, and washed several times with anhydrous ethanol and deionized water, and the black powder obtained was dried at 60 °C for 12 h and denoted as 1T-2H MoS_2_.

PPy@1T-2H MoS_2_ was prepared via in situ chemical polymerization. First, 0.5 g of the obtained 1T-2H MoS_2_ was ultrasonically dispersed in 40 mL of 1.0 M HCl solution. The condensate circulation device was connected to a double beaker, and the temperature was set to 5 °C in an ice water bath. Then, 0.5 mL of pyrrole monomer was added to the hydrochloric acid solution containing 1T-2H MoS_2_ after sonication and transferred to the double beaker. Magnetic stirring was always performed. After 2 h of reaction, the product was filtered and rinsed several times with distilled water. The product was labeled as PPy@1T-2H MoS_2_ pellets, dried at 60 °C for 12 h under a vacuum, and then ground to a powder.

### 2.3. Preparation of the 1T-2H MoS_2_ and PPy@1T-2H MoS_2_ Membranes

First, 0.25 g of PVA particles and 1 g of PVDF powder were added to a round-bottom flask containing 15 mL of dimethyl sulfoxide solvent and stirred for 1 h at 90 °C in a water bath to obtain a brown PVA/PVDF mixture, which was then cooled to room temperature. Then, 0.4 g 1T-2H MoS_2_ and 0.4 g PPy@1T-2H MoS_2_ powders were separately added into two portions of the PVA/PVDF co-mix (5 mL) and stirred for 30 min to obtain the homogeneously mixed solutions. Subsequently, the solutions were transferred to glass plates with a dropper, and the uniform composite films were pushed out on an automatic film applicator. Then, they were quickly placed into water, removed, and dried naturally for 24 h. Finally, the 1T-2H MoS_2_-PVA+PVDF and PPy@1T-2H MoS_2_-PVA+PVDF membrane samples were cut into 33.5 mm diameter circles.

### 2.4. Methods and Characterization

X-ray diffraction (XRD) analysis of the catalysts was conducted in the 2θ range of 10–90° at 40 mA and 40 kV with Bruker D8-advance. The X-ray photon spectra (XPS, JEOL, JPS-9200, Tokyo, Japan) with Mg Kα radiation were employed to collect the X-ray photoelectron spectra of the catalysts. A Bunko-Keiki M30-TP-M device (Tokyo, Japan) was used for the Raman spectroscopy, and the polychromator was excited using a YVO_4_ 532 nm laser. The specimens were heated to 1000 °C at a constant nitrogen flow rate of 50 mL/min. Transmission electron microscopy (TEM, JEOL-2010F, Tokyo, Japan) and scanning electron microscopy (SEM, JEOL JSM-6510LA, Tokyo, Japan) were used to perform the micromorphological and structural analyses. High-angle annular dark-field scanning transmission electron microscopy (HAADF-STEM) images and EDS mapping images were performed on an FEI Titan3 G2 80–300 microscope (Thermo Fisher Scientific, Hillsboro, OR, USA) with an acceleration voltage of 300 kV. The absorbance of the catalysts was tested using a Shimadzu UV-2600 UV-V spectrophotometer(Shidadzu, Suzhou, China).

### 2.5. Fenton-like Catalytic Experiments

Visible photocatalytic reaction tests were performed in a glass reactor with circulating cooling water, using a 300 W iodine tungsten lamp as the light source with a 410 nm cut-off filter and MB as the degradation target. In each photocatalytic degradation experiment, 50 mg of catalyst powder was mixed into 200 mL MB solution (25 mg/L) to obtain a suspension. Before the light reaction, the suspensions were placed in a dark environment under magnetic stirring for 30 min to reach an adsorption–desorption equilibrium. After starting photoluminescence, magnetic stirring was maintained, and 4 mL samples were obtained every 5 min and then centrifuged at 8000 rpm for 5 min. Finally, the absorbance of the MB contaminant was measured at a fixed wavelength of 664 nm using a UV–vis spectrophotometer.

The absorbance value of the degraded solution was measured using the rate of MB decolorization, which was obtained by measuring the absorbance of the degraded solution and extrapolating the rate of degradation. This theory is based on the Lambert–Beer law according to

(1) $$A=\mathrm{lg}(\frac{1}{T})=\mathrm{KlC}\;,$$

where *T* is the ratio of the transmitted light intensity to the incident light intensity available for transmittance, *A* is the relative absorbance, *l* is the thickness of the test solution, *K* is the coefficient of the proportionality of absorbed light, and *C* is the concentration of the organic pollutant. In the equation, *K* and *l* are relatively fixed, while *A* is proportional to *C*; thus, the concentration of the solution will be proportional to the absorbance. Therefore, the degradation rate *D* can be calculated using the following equation:

(2) $$D=\frac{\left(C_{0}\;-\;C_{t}\right)}{C_{0}}=\frac{\;(A_{0}\;-\;A_{t})}{A_{0}},$$

where *C*_0_ represents the initial concentration; *A*_0_ is the initial absorbance; and *C_t_* and *A_t_* represent the concentration and absorbance after light time and the concentration and absorbance after some time *t*, respectively. Factors that affect the rate of the photocatalytic process include the pH of the solution and the initial concentration; thus, experiments were carried out to compare the photocatalysts, controlling for other conditions, such as the oxygen content in the solution, light intensity, and temperature. A comparative experiment was carried out on the photocatalysts, controlling for all other conditions.

The recyclability of the catalyst was investigated. In the cyclic stability experiment, a fixed ratio of catalysts/pollutant/H_2_O_2_/Fe^2+^ was maintained to eliminate the effect of catalyst loss during the recovery process, and all the experiments were repeated three times under the same conditions to eliminate a chance difference from a single experiment.

### 2.6. Solar Water Evaporation Experiment

Solar photothermal evaporated water experiments were conducted in an indoor environment at a room temperature of about 28 °C and relative humidity of about 40%. First, the membranes were placed in a weighing bottle (size of 40 × 25 mm, inner diameter of 35 mm) containing 25 mL of deionized water and moistened for 2 min (floating on the water surface) to ensure that the upper surface was completely wetted. Then, the solar interfacial vapor generation device was placed on an electronic balance under the irradiation of a solar light simulator (adjusted to simulate sunlight using an AM 1.5 filter). The amount of steam generated was calculated by the change in the value displayed on the electronic balance every 10 min. In addition, we obtained the water evaporation performance under different light intensities, where the power density of the solar light simulator was calibrated to 1, 2, and 4 kW/m^2^ using an optical power meter, and the vapor production data of the pure water under dark conditions and one simulated solar light were also measured for the comparison experiment. The photothermal conversion efficiency was calculated using Equation (3) [39]:

(3) $$\eta =\frac{m_{loss}\left(C\times \Delta T+H_{e}\right)}{A\cdot t\cdot C_{opt}\cdot q_{i}}$$

where *η* is the photothermal conversion efficiency, *m_loss_* indicates the amount of mass lost in the system (i.e., the amount of escaped vapor), *A* is the projected area of the solar absorbing layer in the vapor generating device (i.e., the light field formed at the interface when the sunlight was irradiated vertically), *t* is the irradiation time of the sunlight, *C* is the value of the specific heat capacity of water (4.18 J·g^−1^·K^−1^), Δ*T* is the temperature change of the water, *C ×* Δ*T* is the sensible heat, *H_e_* is the latent heat of vaporization of the water (this value changes with temperature), *C_opt_* is the light concentration, and *q_i_* is the optical density of sunlight (1 kW/m^2^). Another important parameter was the photothermal conversion rate *v*, which reflects the escape rate of water vapor in the entire device, and can be calculated using Equation (4) [40]:

(4) $$v=\frac{m_{loss}}{A\cdot t}$$

## 3. Results

### 3.1. Phase Structures of 1T-2H MoS_2_ and PPy@1T-2H MoS_2_

**Figure 1:**
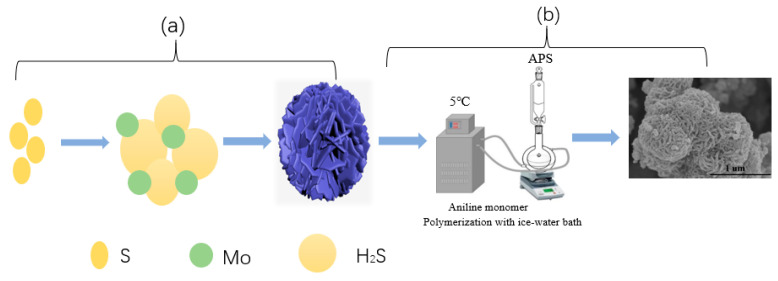
The schematic diagram of the 1T-2H MoS_2_ hydrothermal growth mechanism (**a**) and the PPy@1T-2H MoS_2_ catalyst preparation method (**b**).

Figure 1 illustrates the potential hydrothermal growth mechanism of agglomerated spherical MoS_2_, along with the in situ polymerization of PPy. During the hydrothermal formation of MoS_2_, the presence of propionic acid led to the hydrolysis of thiourea, generating H_2_S. These H_2_S bubbles clustered together, forming a cloud of bubbles with varying sizes. Meanwhile, molybdate ions adsorbed onto the surfaces of these H_2_S bubbles. As the reaction occurred at the gas–liquid interface and ceased when the H_2_S in the bubbles was consumed, this resulted in the formation of agglomerated MoS_2_ microspheres. We propose that the H_2_S bubbles generated through thiourea hydrolysis not only served as the sulfur source for MoS_2_ formation but also acted as structural templates for the nucleation and growth of spherical MoS_2_. The resulting spherical MoS_2_ exhibited a dense structure with smooth surfaces, cross-sections, and a minimal lamellar structure. Part (b) of Figure 1 illustrates the subsequent low-temperature polymerization step to achieve uniformly coated PPy nanoflowers.

**Figure 2:**
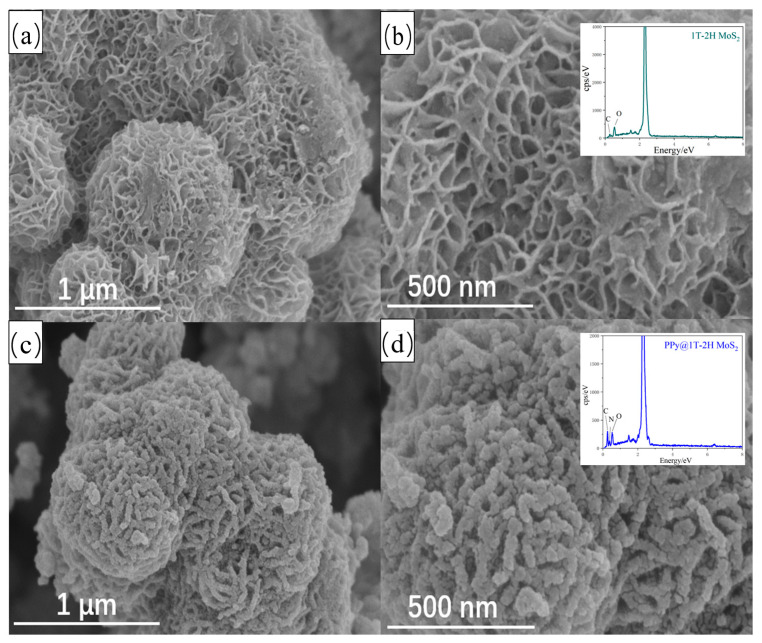
The SEM results of (**a**,**b**) the 1T-2H MoS_2_ and (**c**,**d**) the PPy@1T-2H MoS_2_ with inserts of EDS spectrum.

Figure 2 provides a comparison of the SEM images of 1T-2H MoS_2_ and PPy@1T-2H MoS_2_. Based on the SEM images, it can be observed that the introduction of PPy did not alter the morphology of 1T-2H MoS_2_. Both the 1T-2H MoS_2_ and PPy@1T-2H MoS_2_ consisted of aggregated nanosheets and exhibited a spherical morphology. The diameter of the 1T-2H MoS_2_ nanospheres was approximately 600 nm (Figure 2a), while the PPy@1T-2H MoS_2_ nanospheres had a slightly larger diameter of approximately 700 nm (Figure 2d). Furthermore, Figure 2d clearly depicts a thin and uniform PPy layer wrapping around the edges of the nanoflowers. The presence of the N element in the EDS spectrum further confirms the encapsulation of PPy on the surface of 1T-2H MoS_2_.

**Figure 3:**
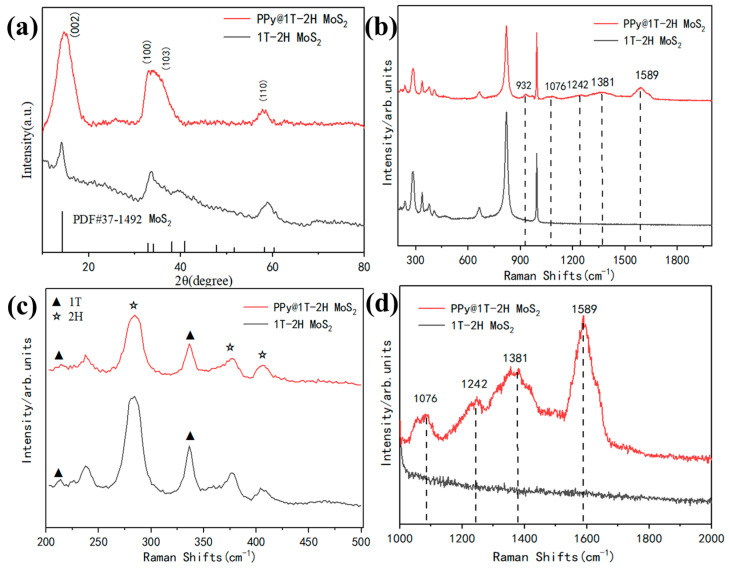
(**a**) XRD patterns of the as-prepared 1T-2H MoS_2_ and PPy@1T-2H MoS_2_; (**b**) a comparison of Raman spectra of 1T-2H MoS_2_ and PPy@1T-2H MoS_2_; (**c**,**d**) the enlarged views of the local areas of the Raman spectra in (**b**).

The composition and crystallinity of a catalyst play crucial roles in determining its photocatalytic performance. XRD analysis was conducted to evaluate the prepared samples (1T-2H MoS_2_ and PPy@1T-2H MoS_2_). The diffraction curve shown in Figure 3a exhibits distinct peaks at 14.3°, 33.9°, 32.3°, and 58.4°, corresponding to the (002), (103), (100), and (110) crystal planes of MoS_2_ (JCPDS 37-1492) [41]. The (002) peak primarily originated from X-ray scattering by the Mo atoms between the MoS_2_ layers, reflecting the number of layers stacked along the c-axis and the interlayer spacing in the MoS_2_ structure. Changes in the number of layers were closely related to the half-height width of the (002) peak [41], while shifts in the peak position indicated alterations in the distance between the MoS_2_ layers. The peak intensities of (100) and (110) also indicated the crystallization of MoS_2_ within the plane. As depicted in Figure 3a, PPy@1T-2H MoS_2_ exhibited a sharp diffraction peak, signifying a good crystallinity. Encouragingly, the encapsulation of PPy on the surface of MoS_2_ was expected to enhance the electron transfer and improve the photocatalytic performance [42].

Figure 3b,c compare the Raman spectra of the 1T-2H MoS_2_ and PPy@1T-2H MoS_2_, which exhibit the characteristic peaks of 2H-phase MoS_2_ at 284 cm^−1^, 376 cm^−1^, and 403 cm^−1^, as well as the peaks of the 1T-phase MoS_2_ peaks at 212 cm^−1^ and 337 cm^−1^ [43]. These peaks arose from the distorted octahedral coordination spheres with a 2a0 × a0 basal superlattice [44], indicating the successful fabrication of 1T-2H MoS_2_ in the catalysts. The presence of PPy did not disrupt the 1T-2H MoS_2_ structure. Furthermore, Figure 3d displays the Raman shifts attributed to PPy, with the peak at 1589 cm^−1^ arising from C=C skeleton stretching. The peaks at 1242 cm^−1^ and 932 cm^−1^ correspond to asymmetric C–H in-plane bending and the in-plane C–H deformation peak, respectively [44,45].

**Figure 4:**
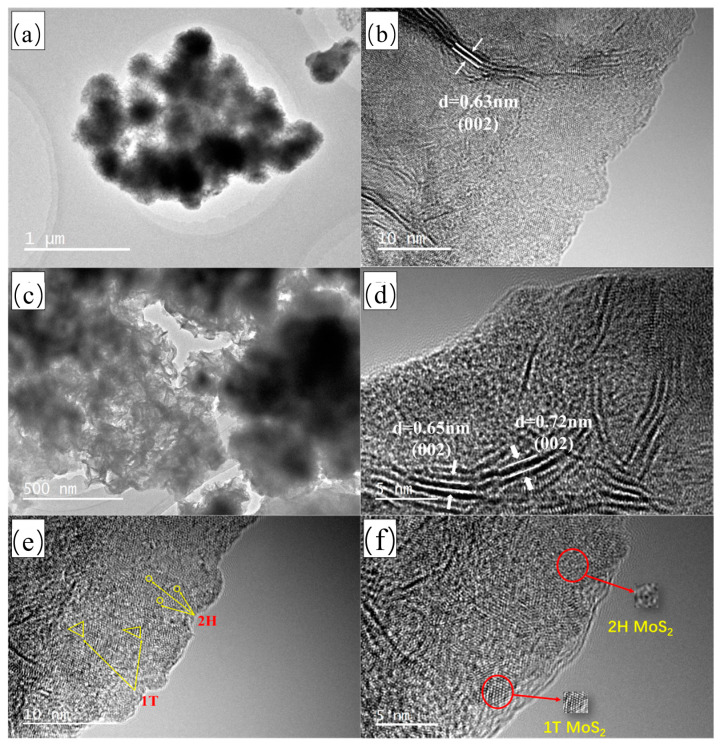
TEM images of (**a**,**b**) 1T-2H MoS_2_ and (**c**,**d**) PPy@1T-2H MoS_2_; HRTEM images of the in-plane (**e**) 1T-2H MoS_2_ and (**f**) PPy@1T-2H MoS_2_ heterostructures showing the crystalline edge spacing of the 1T and 2H phases.

Figure 4a,c display TEM images of the 1T-2H MoS_2_ and PPy@1T-2H MoS_2_, respectively. This reveals that all the prepared samples exhibited a flower-like morphology with a multilayer structure. The corresponding high-resolution image of 1T-2H MoS_2_ is shown in Figure 4b, where a lattice spacing of 0.63 nm corresponding to the (002) crystal plane of MoS_2_ is observed and labeled. Figure 4d–f present the HRTEM images of the PPy@1T-2H MoS_2_ nanocomposites. Interestingly, as depicted in Figure 4d, the interlayer distance of 1T-2H MoS_2_ expanded to 0.72 nm from 0.63 nm due to PPy polymerization. The electrostatic action facilitated the polymerization of PPy layers onto the 1T-2H MoS_2_ nanosheets, weakening the van der Waals forces [44]. The interaction between 1T-2H MoS_2_ and PPy not only resulted in a looser and rougher PPy morphology but also increased the layer spacing of the MoS_2_ nanosheets, which is beneficial for achieving a higher material-specific HRTEM. Figure 4e,f reveal the hexagonal structure with a triangular prismatic arrangement for 2H (Figure 4e) and a triangular or herringbone chain structure with an octahedral arrangement for 1T (Figure 4f), confirming the successful synthesis of the heterogeneous phase 1T-2H MoS_2_ nanosheets. Additional high-angle annular dark-field (HAADF) imaging, as well as STEM-EDS elemental analysis (Figure S1a,b), demonstrates the homogeneous distribution of Mo, S, C, and N elements through the HAADF mapping images, further confirming that PPy was uniformly wrapped around the surfaces of the 1T-2H MoS_2_ nanosheets. The presence of the N element and the increased C element in Table S1 also support the above results.

**Figure 5:**
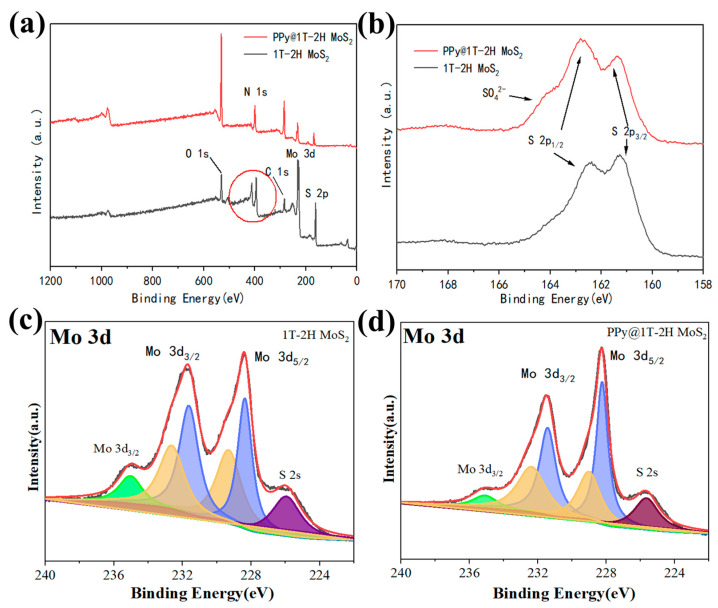
(**a**) The XPS wide survey of 1T-2H MoS_2_ and PPy@1T-2H MoS_2_; M_O_ 3d XPS spectra of (**b**) 1T-2H MoS_2_ and (**c**) PPy@1T-2H MoS_2_. (**d**) Comparison of S 2p spectra of the 1T-2H MoS_2_ and PPy@1T-2H MoS_2_ samples.

XPS analysis was conducted to further investigate the chemical compositions of the in-plane 1T-2H MoS_2_ and PPy@1T-2H MoS_2_ heterostructures. Figure 5a illustrates the presence of Mo, S, C, and O elements, both in the 1T-2H MoS_2_ and PPy@1T-2H MoS_2_, with an additional detection of N in the PPy@1T-2H MoS_2_ due to PPy involvement. Detailed information about the structure and composition was obtained from the Mo 3d and S 2p XPS spectra. Figure 5b,c present the Mo 3d XPS spectra of the 1T-2H MoS_2_ and PPy@1T-2H MoS_2_, respectively. The deconvolution of the Mo 3d peaks revealed four peaks at 231.6, 228.2, 234.6, and 228.9 eV, corresponding to Mo^4+^ 3d_3/2_ and 3d_5/2_ in the 1T phase and Mo^4+^ 3d_3/2_ and 3d_5/2_ in the 2H phase (Figure 5c) [46,47,48,49]. The binding energy of the new peaks in the 1T phase was 0.4 eV lower than that of the corresponding peaks in the 2H phase, which aligns with a previous report [50]. The emergence of these new XPS peaks in the 1T-2H MoS_2_ phase suggests a shift in the Fermi level, indicating the population of additional electrons in the d orbitals during a partial phase transition. Furthermore, the presence of peaks at 232.0, 228.4, 235.0, and 229.3 eV in the 1T-2H MoS_2_ spectrum indicates the 1T phase contribution (Figure 5b). The high-resolution spectrum of S 2p depicted in Figure 5d displays peaks at 162.8 eV and 161.4 eV, corresponding to the S 2p_1/2_ and S 2p_3/2_ orbitals of S^2−^. Additionally, a new S peak at 164.5 eV is observed in the S XPS spectrum of PPy@1T-2H MoS_2_, which can be attributed to the incorporation of SO_4_^2−^ from APS during polymerization. Consequently, the binding energies of both peaks were slightly higher than those of the standard 1T-2H MoS_2_ (162.4 and 161 eV) due to the stronger interaction between 1T-2H MoS_2_ and PPy [51]. The close bond formed through covalent bonding between the two materials reduces the contact resistance and facilitates efficient carrier transport.

Diffuse reflectance UV–vis DRS spectroscopy (Figure S2) was employed to analyze the light absorption properties of the synthesized 1T-2H MoS_2_ and PPy@1T-2H MoS_2_ composites. The absorption of light was observed in the UV–vis region, extending up to 700 nm, indicating their capability to absorb visible light [52]. This enhanced light absorption can be attributed to the presence of PPy wrapping around PPy@1T-2H MoS_2_. Consequently, the PPy@1T-2H MoS_2_ composite exhibited a slight increase in absorbance compared with PPy. The bandgaps of PPy@1T-2H MoS_2_ and 1T-2H MoS_2_ were determined to be 1.45 eV and 1.56 eV, respectively, as illustrated in the figure.

The specific surface area and porosity of the prepared materials were evaluated using N2 adsorption–desorption tests. Figure S3 demonstrates that the BET specific surface area of PPy@1T-2H MoS_2_ (16.8 m^2^/g) exceeded that of 1T-2H MoS_2_ (7.6 m^2^/g). The presence of the PPy coating substantially contributed to the increased BET specific surface area, revealing a layered porous structure with an average pore size of 1.5 nm. Moreover, the pyrrole coating resulted in a significant enlargement of the pore size. This porous structure enhanced the specific surface area and offered abundant active sites for catalytic reactions, facilitating reactant transfer and promoting catalytic processes.

### 3.2. Photocatalytic Activity of the Catalysts

#### 3.2.1. Effect of Catalyst Dosage

**Figure 6:**
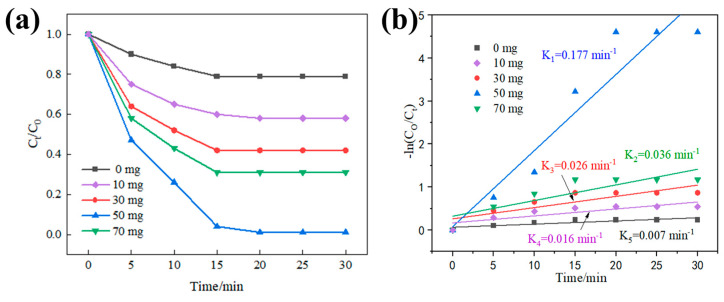
(**a**) MB degradation curves with different PPy@1T-2H MoS_2_ additions and corresponding experimental conditions of 500 μL of H_2_O_2_ in 200 mL of contaminated water ([MB] = 25 mg/L) without Fe^2+^ addition; (**b**) pseudo-first-order kinetic fit for MB photodegradation under simulated solar irradiation.

The photo-Fenton degradation performance of the synthesized materials is investigated using a simulated contaminant MB solution. In Fenton reactions, the initial dose or concentration of the H_2_O_2_ decomposer or metal ions in the reaction solution plays a crucial role in the kinetics of the Fenton reaction. Figure 6a illustrates the impact of the catalyst dosage (0–70 mg) on the efficiency of MB removal in a reaction system with pH = 6 and 500 μL H_2_O_2_ addition in the absence of Fe^2+^ ions. It can be observed that the decolorization of the MB solution significantly improved as the catalyst dosage increased from 0 to 70 mg. With an increase in the reaction time, the residual MB concentration steadily decreased, reaching a stable level after 15 min. Furthermore, the rate constant curve of the reaction was obtained by simulating the kinetics of the MB decolorization process, as depicted in Figure 6b. The complete system exhibited the highest degradation efficiency when the addition of PPy@1T-2H MoS_2_ was 50 mg, with a degradation rate constant of 0.177 min^−1^, which was 5.5 times higher than that of the pure Fenton reaction. The rate constants of PPy@1T-2H MoS_2_ at 10, 30, and 70 mg were 0.016, 0.026, and 0.036 min^−1^, respectively, and these values were much lower than the rate at 50 mg of PPy@1T-2H MoS_2_. This indicates that the catalyst concentration in the reaction system significantly affected the removal efficiency of MB. This can be attributed to the fact that 70 mg of PPy@1T-2H MoS_2_ catalyzed the generation of an excess of ·OH from H_2_O_2_, as depicted in Equation (10). The possible recombination of ·OH with hydrogen peroxide resulted in the production of H_2_O_2_ and ·O_2_H, which limited the cycling of the Fenton reaction [53].

**Figure 7:**
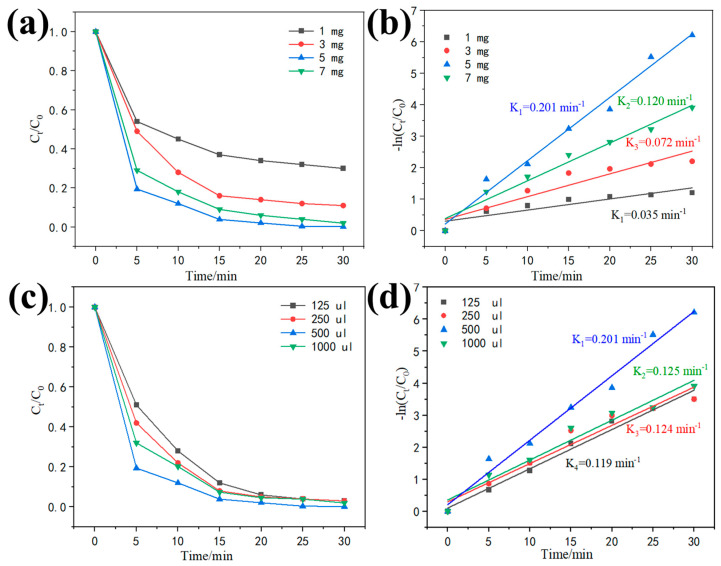
Degradation curves of MB at different (**a**) FeSO_4_ (50 mg of catalyst and 500 μL of H_2_O_2_ at pH = 6 and 30 min of reaction time) and (**c**) H_2_O_2_ additions (50 mg of PPy@1T-2H MoS_2_ catalyst and 5 mg FeSO_4_ in contaminated water ([MB] = 25 mg/L)); (**b**,**d**) pseudo-first-order kinetic fit of each condition to simulate the photo-degradation of MB under solar irradiation.

The effect of Fe^2+^ addition on the MB removal efficiency is illustrated in Figure 7a. After adding 50 mg of catalyst and 500 μL of H_2_O_2_ at pH = 6 with a reaction time of 30 min, it was observed that the degradation efficiency of MB increased with the addition of Fe^2+^. However, when the Fe^2+^ addition exceeded 5 mg, the degradation efficiency started to decrease. This decrease in efficiency could be attributed to the excessive addition of Fe^2+^, leading to the reduction of H_2_O_2_ and resulting in an overabundance of the ·OH radical. It is significant to note that H_2_O_2_ serves as the source of **·**OH production and plays a crucial role in the Fenton reaction, influencing the kinetics of organic pollutant degradation. Figure 7c demonstrates the effect of hydrogen peroxide dosage on the decolorization of MB. The initial H_2_O_2_ dosage was adjusted from 125 μL to 1000 μL, and the reaction time for MB degradation was measured. It was observed that insufficient H_2_O_2_ dosage resulted in low MB removal efficiency, as the production of ·OH radicals was not sufficient [54,55]. When the H_2_O_2_ dose was 500 μL, the degradation effect reached 99.8% within 30 min. However, increasing the H_2_O_2_ dosage to 1000 μL did not significantly enhance the removal efficiency. This could be attributed to a larger concentration of hydroxyl radicals generated at the beginning of the reaction due to the increased H_2_O_2_ dosage in the system [53]. Subsequently, the excess hydrogen peroxide inhibited the active sites on the surface of PPy@1T-2H MoS_2_. Therefore, determining the optimal dose of the Fenton reagent for the removal of organic contaminants is crucial to minimize the process cost.

The impact of the initial solution pH on the removal efficiency of MB was examined within a pH range of 2 to 6. Figure S4a illustrates that the pH had a noticeable effect on the color removal efficiency. By the end of the 30 min reaction, all pH values achieved approximately 99% removal efficiency. After a 5 min reaction period (Figure S4b), the degradation efficiencies vary across different pH levels: 79.7% for pH 2, 91.7% for pH 3, 95.9% for pH 4, 90% for pH 5, and 80.6% for pH 6. The highest efficiency was observed at pH 4. Acidic conditions were conducive to the degradation of H_2_O_2_ in the Fenton reaction, as H^+^ ions play a vital role in the generation of ·OH, thereby enhancing the overall efficiency of the Fenton reaction [56]. The graph reveals that the catalytic effect was lower at pH 2 compared with other pH values due to the hindered Fe catalytic reaction at a low pH and the facile oxidation of organic matter under strong acidic conditions. Extremely low pH not only inhibited the reaction but also led to the precipitation of Fe as iron hydroxide, resulting in a loss of catalytic capacity in the solution [57].

#### 3.2.2. Catalytic Activity of Hybrid Nanoparticles

**Figure 8:**
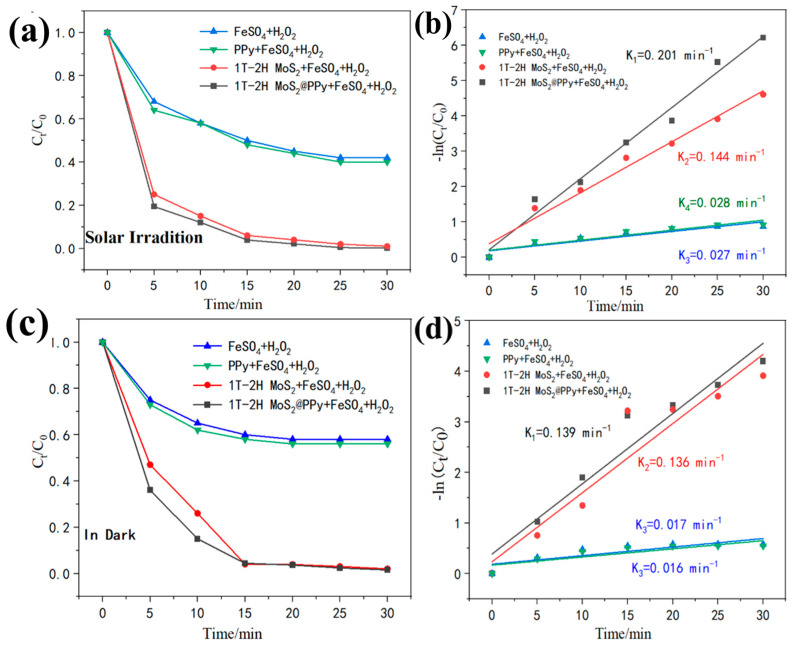
(**a**,**c**) Degradation efficiency of the traditional Fenton method, pure PPy, 1T-2H MoS_2_, and PPy@1T-2H MoS_2_ catalysts for MB, where the experimental conditions were 50 mg of catalyst, 5 mg of FeSO_4,_ and 500 μL of H_2_O_2_ in 200 mL of contaminated water ([MB] = 25 mg/L); (**b**,**d**) pseudo-first-order kinetic fit of each to simulate the photodegradation of MB under solar irradiation and in the dark.

To assess the photo-Fenton degradation capabilities of the synthesized materials, we conducted degradation experiments using MB dye as the contaminant under simulated light and dark reaction environments. Figure 8a,c compare the degradation effects of PPy@1T-2H MoS_2_, 1T-2H MoS_2_, PPy, and the conventional Fenton method (H_2_O_2_-Fe^2+^) on MB in both light and dark conditions. It is observed that both 1T-2H MoS_2_ and PPy@1T-2H MoS_2_ catalysts exhibited excellent pollutant removal abilities under both light and dark conditions, rapidly degrading the simulated pollutant within the first 15 min of the reaction (Figure 8). 

Furthermore, PPy@1T-2H MoS_2_ demonstrated superior degradation performance compared to 1T-2H MoS_2_, regardless of the presence or absence of light. This observation suggests a synergistic effect between PPy and 1T-2H MoS_2_ in the PPy@1T-2H MoS_2_ composite, which accelerated the degradation of organic matter. This synergistic effect can be attributed to the unique band structure provided by PPy doping, particularly the presence of different band gap energies, which enhanced the photo-Fenton catalytic activity. Additionally, when comparing PPy@1T-2H MoS_2_ with the conventional Fenton method and PPy alone, it is evident that the degradation efficiency of the PPy@1T-2H MoS_2_ system for simulated organic compounds was significantly higher than that of the conventional Fenton reaction in both reaction sceneries, as depicted in Figure 8. This indicates that PPy@1T-2H MoS_2_ exhibits a synergistic effect with the Fenton reaction reagent, facilitating the decomposition of organic matter.

Figure S5 presents the TOC analysis of MB during photocatalytic degradation using various catalysts, including the conventional Fenton method, pure PPy, 1T-2H MoS_2_, and PPy@1T-2H MoS_2_. As shown in the figure, the degradation efficiency of PPy@1T-2H MoS_2_ remains high, reaching up to 83.5% even after multiple repetitions under light conditions. This was the highest efficiency observed among all the samples. It is important to point out that the TOC degradation effect in this figure, approximately 80%, is not as high as those shown in Figure S4 and Figure 8. This difference could be attributed to the organic carbon present in the PPy@1T-2H MoS_2_, which may have been released into the solution during the degradation process.

**Figure 9:**
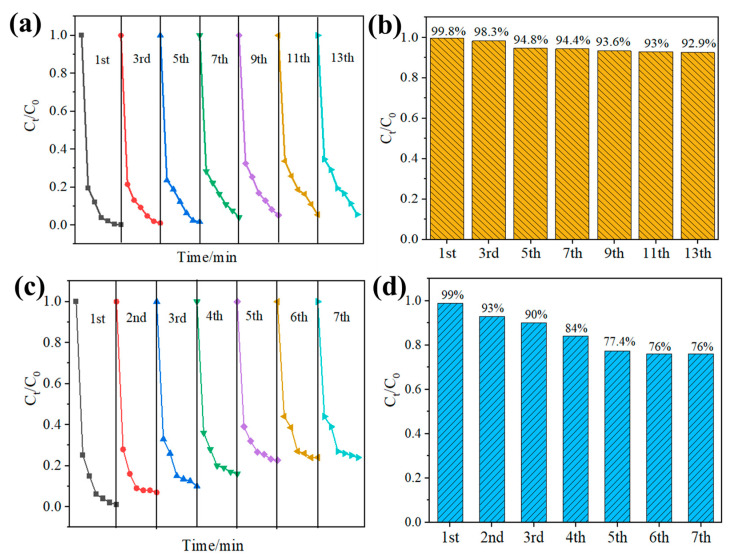
Re-usability test for photocatalytic degradation of MB by PPy@ 1T-2H MoS_2_ (**a**,**b**) and 1T-2H MoS_2_ under solar irradiation (**c**,**d**).

The recyclability and structural stability of the catalyst are crucial factors for evaluating its effectiveness and economic viability. Therefore, the reversibility of PPy@1T-2H MoS_2_ for MB degradation was investigated. Multiple runs were conducted to assess the reusability of PPy@1T-2H MoS_2_ for MB removal, with each run starting with an initial MB concentration of approximately 25 mg/L. Figure 9a–d illustrates the trend of MB removal efficiency over successive runs. It can be observed that the removal of MB decreased as the number of runs increased. In the case of the PPy@1T-2H MoS_2_ reaction system (Figure 9b), a slight decrease in MB removal is observed with increasing runs, ranging from 99.8% to 92.9% during the first 13 degradation runs. Surprisingly, the PPy-coated 1T-2H MoS_2_ demonstrated superior stability during cycling compared with 1T-2H MoS_2_ (Figure 9d). The degradation rate of 1T-2H MoS_2_ decreased to 76% by the seventh run, indicating a significant improvement in the cycle stability of the PPy@1T-2H MoS_2_ composites.

In order to confirm the cycling stability of the material and to understand the reason for the degradation of the material, the morphology and XPS of the samples after multiple cycling tests were tested and compared with the results before cycling. Figure S9 shows the morphology of the PPy@1T-2H MoS2 after the fifth cyclic measurement. The spherical morphology is shown in Figure S8, and the composites consisted of aggregated PPy-coated nanosheets. It can be observed that the cyclic measurement did not alter the morphology of PPy@1T-2H MoS_2_, indicating the good structure stability of the composites.

Although the exact reason is not fully understood, it is evident that the 1T phase in MoS_2_ was inherently unstable, and under the influence of light, the 1H phase in the hybrid material transformed into the 2H phase, resulting in a decrease in the catalytic activity of the material [58]. As shown in Figure S9, we compared the Mo3d XPS of the 1T-2H MoS_2_ after the fifth cyclic measurement. The results support the assumption that the content of 1T decreased while the 2H phase content increased. Meanwhile, compared with 1T-2H MoS_2_ (Table S2), the introduction of PPy effectively retarded the transition from the 1T to 2H phase of 1T-2H MoS_2_ during the catalytic cycle. Semiconductor photo-corrosion typically occurs when the semiconductor undergoes oxidation or reduction by photo-generated electrons or holes. This can be effectively mitigated if the photo-generated charges are rapidly transferred from the semiconductor. The coating of PPy on the surface of 1T-2H MoS_2_ might enhance the cyclic stability of the hybrid material by facilitating the efficient transfer of photogenerated charges.

### 3.3. Application of Solar Energy in Water Evaporation

**Figure 10:**
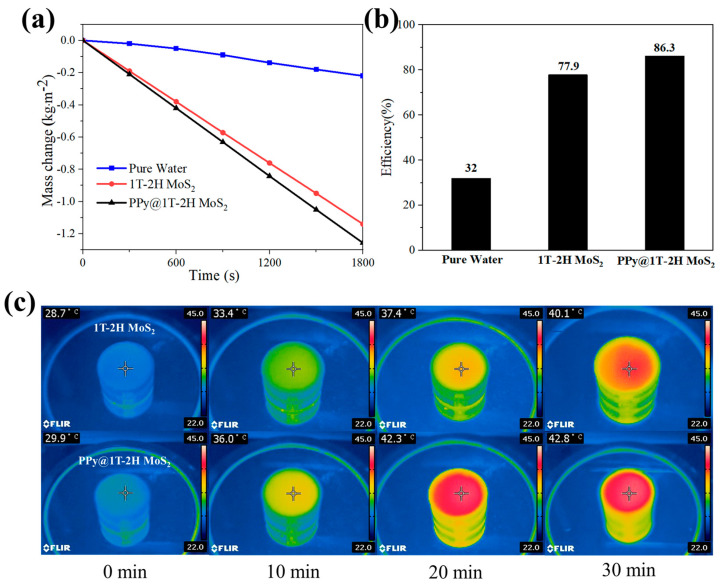
Solar vapor generation performance of the samples prepared under one solar irradiation: (**a**) weight change, (**b**) efficiencies of different materials (pure water, 1T-2H MoS_2_, and PPy@1T-2H MoS_2_), and (**c**) the IR images of the surface temperatures for the 1T-2H MoS_2_ and PPy@1T-2H MoS_2_ evaporation systems.

We investigated the solar vapor performance of the above catalysts, which involved harnessing solar energy for capturing solar radiation using the prepared catalyst. Specifically, we explored the solar water evaporation performance of films fabricated with 0.4 g of 1T-2H MoS_2_ and PPy@1T-2H MoS_2_. Under simulated sunlight with an intensity of 1 kW/m^2^, the photothermal conversion efficiency of 1T-2H MoS_2_ was determined to be 86.3% (Figure 10), which is higher compared with the sample without PPy. The enhancement can be attributed to the presence of PPy, which increased the absorption rate of sunlight for 1T-2H MoS_2_. Consequently, more light energy was converted into heat energy, leading to an accelerated production of solar steam. These findings are consistent with the results obtained from the UV–vis DRS spectra.

Efficient heat management is vital for improving the water evaporation efficiency. To evaluate the thermal convection properties of the 1T-2H MoS_2_ and PPy@1T-2H MoS_2_, temperature changes were measured for different evaporation systems under one solar irradiation. As depicted in Figure 10c, after 30 min of testing, the measured surface temperatures of the two evaporation systems were recorded as 40.1 °C and 42.8 °C. This indicates that the heat generated by 1T-2H MoS_2_ and PPy@1T-2H MoS_2_ was concentrated on the surface, resulting in a rapid increase in temperature from 28.7 °C and 29.9 °C to 40.1 °C and 42.8 °C, respectively. Moreover, the heat converted through solar energy absorption was predominantly directed toward heating the upper part of 1T-2H MoS_2_ rather than dissipating into the surrounding environment (as depicted by the blue-green area in Figure 10c).

## 4. Discussion

### 4.1. The Synergistic Effect of Photocatalytic Degradation and the Fenton Reaction

Based on the findings presented in Figure 6, Figure 7 and Figure 8, it is evident that PPy@1T-2H MoS_2_ exhibited superior photo-Fenton organic degradation ability compared with 1T-2H MoS_2_. Additionally, the degradation efficiency achieved by PPy@1T-2H MoS_2_ surpassed that of the conventional Fenton reaction of H_2_O_2_ + FeSO_4_. The enhanced degradation efficiency of organic pollutants can be attributed to two main factors. First, there was a synergistic effect between PPy and 1T-2H MoS_2_ in the PPy@1T-2H MoS_2_ composites. This synergistic effect amplified the catalytic activity of the material, leading to improved organic degradation. Second, there was a synergistic effect between photocatalytic degradation and the Fenton reaction. The combination of these two mechanisms enhanced the overall degradation process, resulting in higher efficiency in removing organic contaminants.

Therefore, the combination of PPy and 1T-2H MoS_2_ in PPy@1T-2H MoS_2_ not only improved the photocatalytic performance but also enhanced the catalytic activity of the Fenton reaction. This synergistic effect contributed to the remarkable degradation efficiency observed for organic pollutants, surpassing the capabilities of both 1T-2H MoS_2_ and the conventional Fenton reaction [59,60].

**Figure 11:**
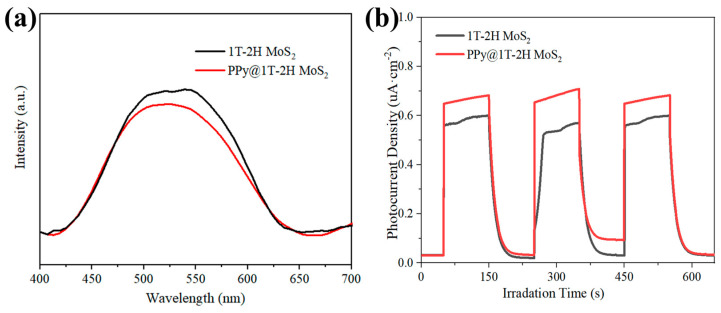
(**a**) Photoluminescence (PL) spectra of 1T-2H MoS_2_ and PPy@1T-2H MoS_2_; (**b**) transient photocurrent densities for different samples.

In order to investigate the synergistic effect between the PPy and 1T-2H MoS_2_, various analyses were conducted and the results are presented in Figure 11. First, the photoluminescence spectra were compared between PPy-polymerized 1T-2H MoS_2_ nanospheres and 1T-2H MoS_2_. In Figure 11a, it can be observed that the photoluminescence intensity of the 1T-2H MoS_2_ nanospheres decreased after PPy polymerization. This reduction in intensity indicates that the complexation of photogenerated electrons and holes was suppressed and reduced to a certain extent. The transient photocurrent density spectrum was analyzed for the PPy-coated 1T-2H MoS_2_ nanospheres, as shown in Figure 11b. The pure 1T-2H MoS_2_ nanosheets exhibited a smaller photocurrent under light conditions, suggesting that the photogenerated electrons and holes easily recombined. However, when PPy was doped with 1T-2H MoS_2_, the photogenerated holes were easily captured by PPy, inhibiting the recombination of photogenerated electrons and holes. As a result, more photogenerated carriers were involved in the conductivity under the lighting conditions used. This observation aligns with the analysis of the photoluminescence spectra. The synergistic effect between PPy and 1T-2H MoS_2_ in the PPy@1T-2H MoS_2_ composite enhanced the separation of photo-generated electrons from holes during the photo-Fenton reaction. This separation reduced the transition from the 1T phase to the 2H phase in MoS_2_, which is prone to photo-corrosion. As a result, the degradation recyclability and structural stability of PPy@1T-2H MoS_2_ were improved, as evidenced by the result presented in Figure 9.

### 4.2. Photothermal Enhanced Photo-Fenton Catalytic Degradation

Based on the results presented in Figure 10, the PPy@1T-2H MoS_2_ composites demonstrated outstanding photothermal properties, achieving an impressive effluent evaporation efficiency of up to 86% when utilized as a solar interfacial water evaporation film. Previous studies highlighted the positive correlation between temperature and Fenton reaction efficiency, with temperature evaluations ranging from 10 to 50 °C significantly enhancing the reaction’s effectiveness [61].

The photothermal properties of PPy@1T-2H MoS_2_ are investigated to understand its role in the photo-Fenton reaction system. Figure S6 compares the infrared (IR) thermal images of the PPy@1T-2H MoS_2_ composite during visible light irradiation. It can be observed that the temperature of the 1T-PPy@2H MoS_2_ composite increased significantly, with a temperature rise of 8 °C after 10 min of irradiation, as shown in Figure S7a, while the pure water showed only a 1.5 °C temperature change. This indicates a reasonable photothermal conversion efficiency of PPy@1T-2H MoS_2_. The photothermal effect induced by the temperature rise can accelerate the degradation of catalytic reagents in the photo-Fenton process. The kinetics constant (k) values presented in Figure S7c for PPy@1T-2H MoS_2_ were increased by 1.4 times, demonstrating obvious photothermal-enhanced photo-Fenton catalysis. This finding is consistent with a study by Shi et al. [62], who reported the use of a photo-Fenton catalyst composed of magnetic CuFe_2_O_4_@MIL-100(Fe, Cu) metal-organic frameworks (MCuFe MOF) for MB degradation. The combination of photothermal conversion and the Fenton reaction synergistically accelerated the degradation of pollutants. Increasing the temperature of the solution can promote the kinetic constants of the Fenton process, particularly in the control step of the Fenton reaction system. Additionally, it was reported that photothermal materials can generate “hot electrons”, which can enhance the separation of photogenerated electron–hole pairs and improve the catalytic activity. These effects contribute to the overall enhancement of the photo-Fenton process [62,63,64].

### 4.3. Photothermal-Fenton Reaction Mechanism

**Figure 12:**
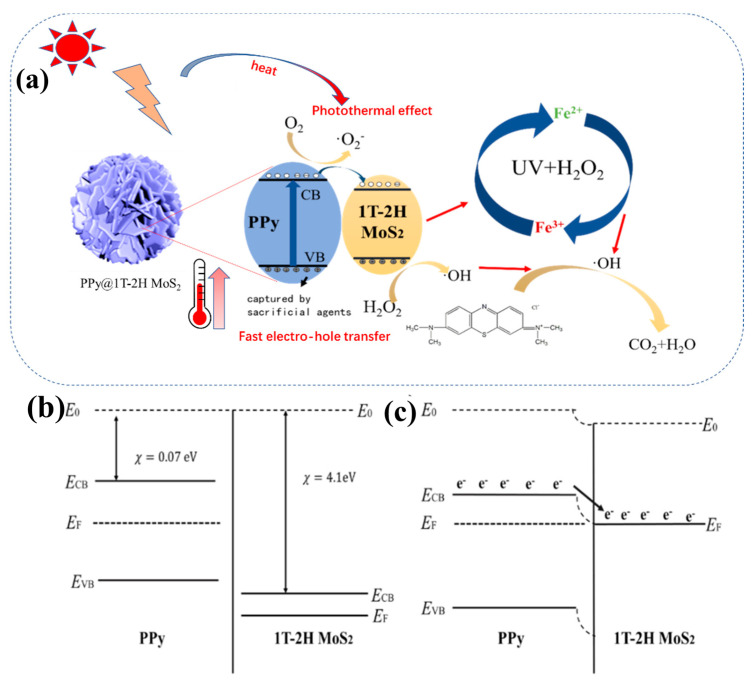
(**a**) The photothermal-Fenton reaction mechanism of PPy@1T-2H MoS_2_. (**b**,**c**) Carrier transport mechanism at carrier interface.

Figure 12a proposes a potential photo-Fenton reaction mechanism to explain the enhanced photocatalytic activity observed in PPy@1T-2H MoS_2_. The introduction of PPy in the composite leads to the hybridization of the energy bands of both semiconductors, resulting in a lower band gap structure of 1T-2H MoS_2_, which promotes the separation of photogenerated electron–hole pairs and increases the generation of hydroxyl radicals (·OH). The enhanced photocatalytic activity and stability of PPy@1T-2H MoS_2_ compared with 1T-2H MoS_2_ are confirmed in Figure 8 and Figure 9.

To further understand the carrier transport mechanism at the interface of PPy@1T-2H MoS_2_, Figure 12b illustrates the energy levels of PPy and MoS_2_. PPy has a higher Fermi energy level than MoS_2_, resulting in electron transfer from PPy to 1T-2H MoS_2_ upon contact until their Fermi levels reach equilibrium. As shown in Figure 12c, after an electron transfer, a thin layer of immobile ionized host is left in the PPy, while an immobile ionized acceptor is created on the 1T-2H MoS_2_. This generates an internal electric field (E) at the interface between PPy and 1T-2H MoS_2_ that points from PPy to 1T-2H MoS_2_. Under visible light illumination, the excited carriers in 1T-2H MoS_2_ can more easily pass through the heterojunction barrier of PPy@1T-2H MoS_2_, resulting in enhanced carrier transport [58,65]. PPy acts as a fast electron trap, limiting the recombination of photogenerated electron-hole pairs (Figure 12c). This facilitates the generation of more hydroxyl radicals (·OH) and enhances the photocatalytic degradation of the com.posite.

The synergistic effect between PPy and 1T-2H MoS_2_ enhances the separation of photogenerated electron–hole pairs, leading to accelerated photocatalytic and photo-Fenton reactions. Additionally, 1T-2H MoS_2_ demonstrates superior organic degradation ability compared with the Fenton reagent in both light and dark reaction environments.

This indicates that 1T-2H MoS_2_ accelerates the decomposition of H_2_O_2_, and the degradation efficiency of PPy@1T-2H MoS_2_ is significantly higher than the conventional Fenton reagent, even in the dark without photocatalytic involvement. In the presence of light, the degradation efficiency of PPy@1T-2H MoS_2_ is significantly higher than that of the conventional Fenton reagent, indicating that the photocatalytic process of PPy@1T-2H MoS_2_ enhances the Fenton reaction under light catalysis. The generated Fe^3+^ species can be regenerated to Fe^2+^ through a series of reactions (Equations (5)–(13)). The transferred electrons and remaining holes in PPy@1T-2H MoS_2_ are likely involved in the generation of hydroxyl radicals (·OH) from H_2_O_2_, thereby accelerating Fe^2+^/Fe^3+^ conversion and enhancing the Fenton reaction. Furthermore, the Mo^4+^/Mo^6+^ species in PPy@1T-2H MoS_2_ can also catalyze the decomposition of H_2_O_2_ to some extent. According to the previous reports [36,39,43,56,57,66], the specific photo-Fenton reaction system is complex and may involve multiple simultaneous reactions as follows:

PPy@1T-2H MoS_2_ + *hv* → e^−^ + h^+^, (5)

e^−^ + O_2_ → O_2_^−^, (6)

H_2_O_2_ + *hv* → 2·OH, (7)

H_2_O_2_ + ·O_2_^−^ → ·OH + OH^−^, (8)

Mo^4+^ + 2H_2_O_2_ → Mo^6+^+2OH^−^ + 2·OH, (9)

Mo^6+^ + 2H_2_O_2_ → Mo^4+^+2·O_2_H + 2H^+^, (10)

Fe^2+^ + H_2_O_2_ → Fe^3+^ + ·OH + OH^−^, (11)

Fe^3+^ + H_2_O_2_ + *hv* → Fe^2+^ + ·O_2_H + H^+^ (12)

OH/OH^−^/h^+^ + MB → CO_2_ + H_2_O. (13)

Furthermore, the PPy@1T-2H MoS_2_ composites exhibit exceptional photothermal activity. The temperature elevation resulting from the enhanced photothermal effect of PPy@1T-2H MoS_2_ (as observed in Figure 10) further contributes to the improved efficiency of the Fenton degradation process. The temperature increase accelerates the generation rate of ·OH radicals, promoting their reaction with organic pollutants and enhancing the oxidation effect and removal rate of chemical demand (COD) in the system, as previously reported [66].

It is important to note that the proposed reactions and mechanisms should be supported by experimental evidence and further studies to fully understand the intricate details of the photo-Fenton process in the PPy@1T-2H MoS_2_ system.

## 5. Conclusions

In this study, we successfully synthesized PPy-coated 1T-2H MoS_2_ nanoflowers as innovative photothermal enhanced photo-Fenton catalysts. The results of the photo-Fenton catalysis results demonstrate that the PPy@1T-2H MoS_2_ nanocatalysts exhibited superior catalytic activity and cyclic durability compared with 1T-2H MoS_2_, as well as outperforming the conventional Fenton reagent in terms of degradation rates. Even after 13 cycles, the degradation efficiency of MB remained above 92% when using the Fenton reaction with PPy@1T-2H MoS_2_. XPS confirmed that the PPy in the hybrid material enhanced the stability of 1T-2T MoS2 by slowing down the transition from the 1T phase to the 2H phase.

The enhanced photo-Fenton degradation of organic pollutants can be attributed to several factors. First, the synergistic effect between 1T-2H MoS_2_ and PPy effectively improved the photocatalytic activity and inhibited the recombination of photogenerated electrons and holes, thereby increasing the stability and overcoming its poor cycling efficiency. Second, the coupling effect of photocatalytic degradation and the photo-Fenton reaction accelerated the degradation of pollutants. Additionally, the PPy@1T-2H MoS_2_ composites exhibited excellent photothermal activity, leading to a temperature rise in the reaction system, which further facilitated the photothermal-promoted Fenton degradation efficiency.

Furthermore, in terms of the solar water evaporation performance, PPy@1T-2H MoS_2_ achieved a remarkable evaporation rate of 1.26 kg/m^2^ in just 30 min, surpassing the performance of 1T-2H MoS_2_. The introduction of PPy significantly enhanced the ability to absorb visible light and improve the catalytic efficiency. These findings hold promising implications for the development of a novel catalyst aimed at eliminating harmful and toxic compounds from the environment.

## Data Availability

The data presented in this study are available on request from the corresponding author. The data are not publicly available due to [Confidentiality requirements].

## Supplementary Materials

The following supporting information can be downloaded at: https://www.mdpi.com/article/10.3390/polym15193900/s1, Figure S1. HAADF-STEM images and element-mapping images of the magnified branched region in the (a) PPy@1T-2H MoS_2_ and (b) 1T-2H MoS_2_ nanospheres; Figure S2. UV–vis spectra of PPy@1T-2H MoS_2_ and 1T-2H MoS_2_; Figure S3. (a) Adsorption–desorption isotherms for N_2_ for PPy@1T-2H MoS_2_ and 1T-2H MoS_2_; (b) pore diameter distribution diagram; Figure S4. (a) Effect of initial pH on the decolorization of MB by Fenton oxidation; (b) degradation efficiency of the reaction for 5 min. The experimental conditions were 50 mg PPy@1T-2H MoS_2_ catalyst, 5 mg of FeSO_4_, and 500 μL of H_2_O_2_ in 200 mL of contaminated water ([MB] = 25 mg/L).; Figure S5 TOC analysis of MB in photocatalytic degradation process by the traditional Fenton method, pure PPy, 1T-2H MoS_2_, and PPy@1T-2H MoS_2_ catalysts. The experimental conditions consisted of 50 mg of catalyst, 5 mg of FeSO_4_, and 500 μL of H_2_O_2_ in 200 mL of contaminated water ([MB] = 25 mg/L); Figure S6 IR images of the temperature change of the upper surface of Water, MB and PPy@1T-2H MoS_2_+MB system in 30 min; Figure S7 (a) Photothermal heating curves of the pure water, MB-containing water, and PPy@1T-2H MoS_2_+MB containing water. (b) Relative concentration changes (C_t_/C_0_) of MB and Photothermal heating curves of the PPy@1T-2H MoS_2_ with and without temperature control. (c) The kinetic fit for MB degradation by PPy@1T-2H MoS_2_ composites at various temperatures; Figure S8 The SEM morphology of the PPy@1T-2H MoS_2_ after the 5th cyclic test; Figure S9. The Mo 3d XPS spectrum of 1T-2H MoS_2_ (a) before the first test and (b) after the 5th cyclitic test; Table S1: Sample elemental composition weight percent ratio; Table S2: Changes in the quantization ratio of 2H to 1T phase in1T-2H MoS_2_ during the cyclitic test.
