# Supplementary material for: An Investigation of PPy@1T/2H MoS2 Composites with Durable Photothermal-Promoted Effect in Photo-Fenton Degradation of Methylene Blue and in Water Evaporation
Source: Polymers (Basel). 2023 Sep 27;15(19):3900. doi: 10.3390/polym15193900 (PMC10575121; doi:10.3390/polym15193900)
Supplement: Supplementary file 1 [file polymers-15-03900-s001.zip › polymers-2570550-supplementary.pdf]

Supplementary Materials

An Investigation of PPy@1T/2H MoS_2 Composites with Durable Photothermal-Promoted Effect in Photo-Fenton Degradation of Methylene Blue and in Water Evaporation

Yanhua Lei [1,*], Da Huo [1], Hui Liu [1], Sha Cheng [2], Mengchao Ding [1], Bochen Jiang [1], Fei Zhang [1,*], Yuliang Zhang [1] and Guanhui Gao [3]

[1] Institute of Marine Materials Science and Engineering, Shanghai Maritime University, Shanghai 201306, China; huoda18852959827@163.com (D.H.); liang.zai.la@outlook.com (H.L.); dingmengchao111@163.com (M.D.); bochenjiang@hotmail.com (B.J.); ylzhang@shmtu.edu.cn (Y.Z.)

[2] Qingdao Product Quality Testing Research Institute, Qingdao 266061, China; xigua5200@163.com

[3] Material Science and Nano engineering Department, Rice University, Houston, TX 77005, USA; gg13@rice.edu

* Correspondence: yhlei@shmtu.edu.cn (Y.L.); zhangfei@shmtu.edu.cn (F.Z.)

Figure S1

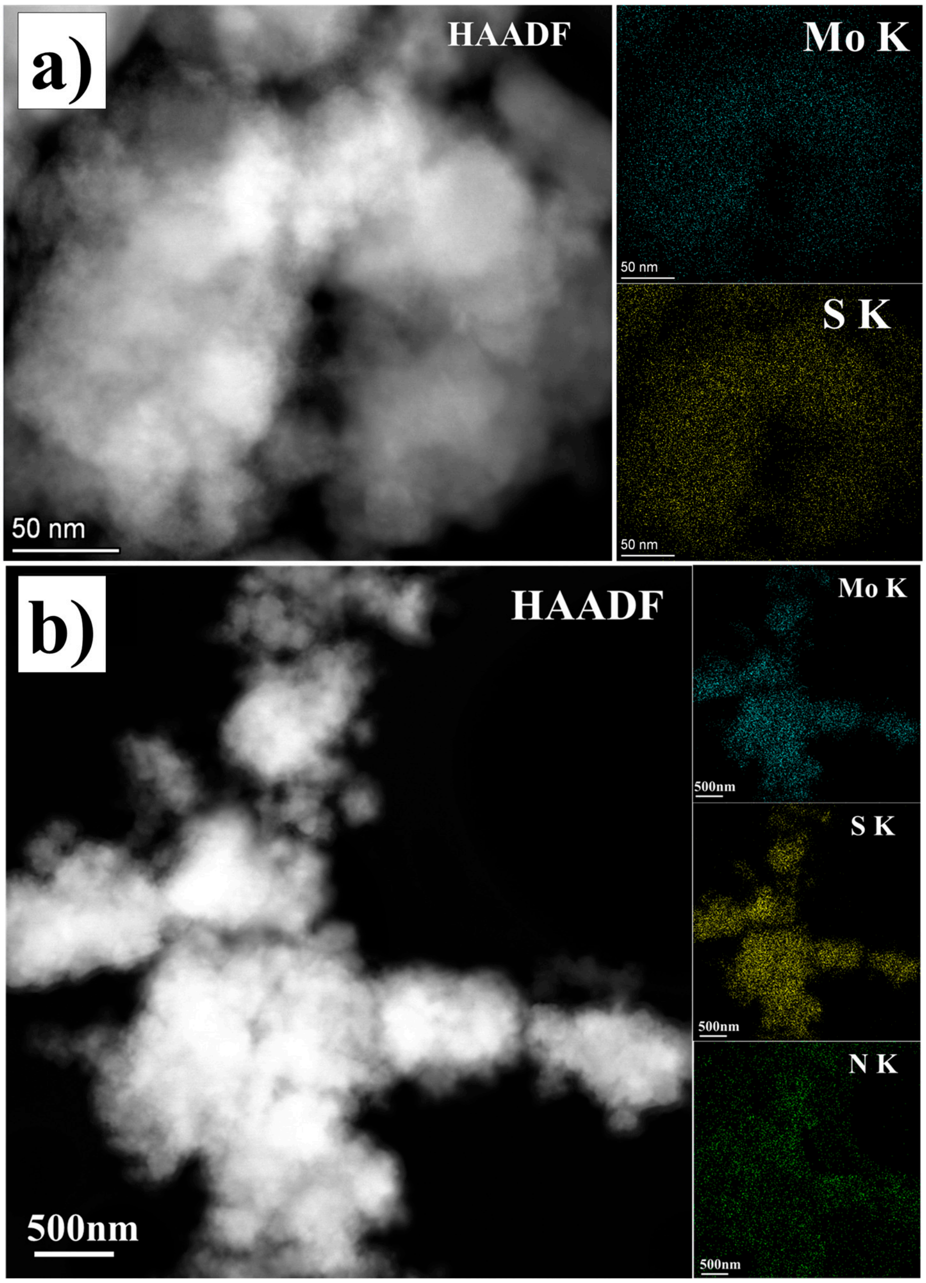

Figure S1. HAADF-STEM images and element-mapping images of the magnified branched region in the(a) PPy@1T-2H MoS_2 and (b) 1T-2H MoS_2 nanospheres.

Figure S2

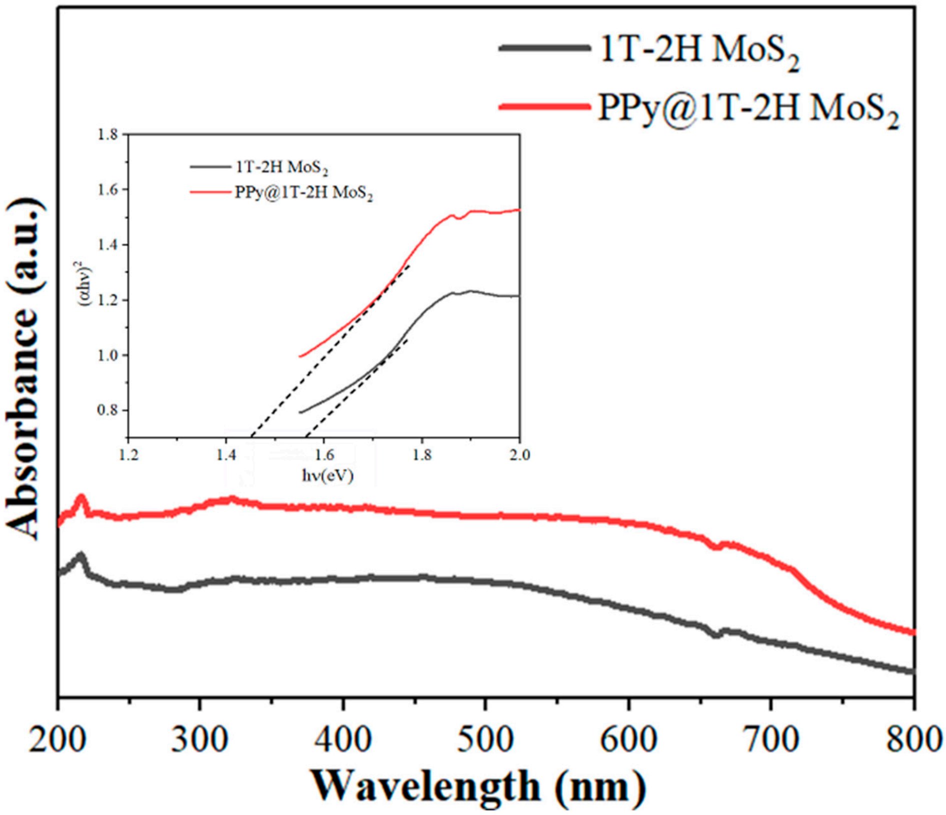

Figure S2. UV–vis spectra of PPy@1T-2H MoS_2 and 1T-2H MoS_2.

Figure S3

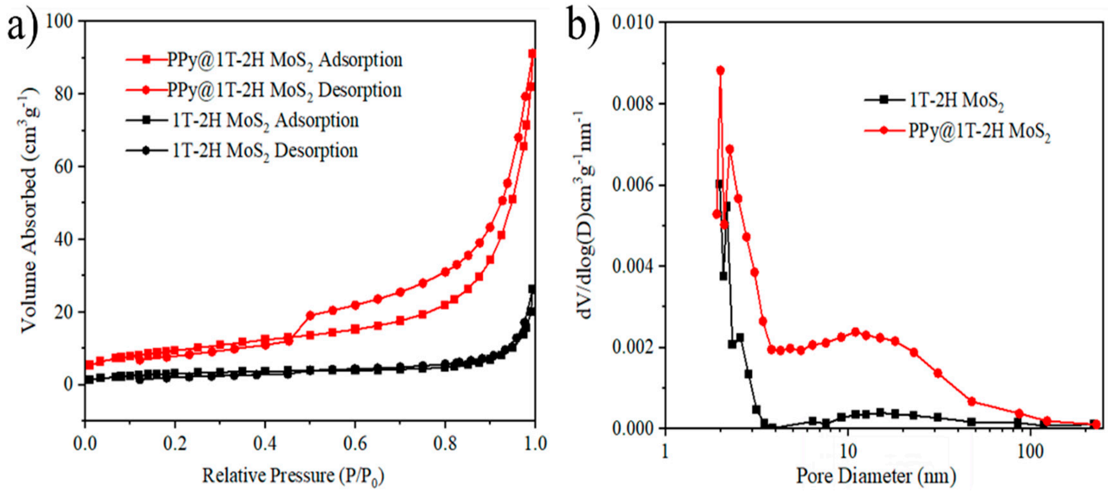

Figure S3. (a) Adsorption–desorption isotherms for N_2 for PPy@1T-2H MoS_2 and 1T-2H MoS_2; (b) pore diameter distribution diagram.

Figure S4

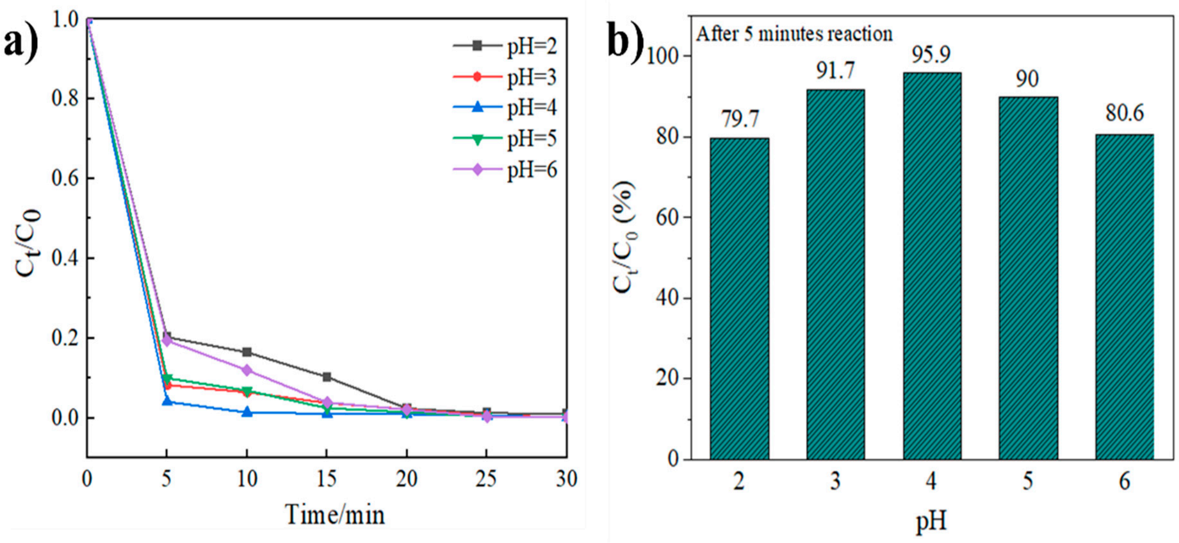

Figure S4. (a) Effect of initial pH on the decolorization of MB by Fenton oxidation; (b) degradation efficiency of the reaction for 5 min. The experimental conditions were 50 mg PPy@1T-2H MoS_2 catalyst, 5 mg of $FeSO_4$, and 500 μL of H_2O_2 in 200 mL of contaminated water ([MB] = 25 mg/L).

Figure S5

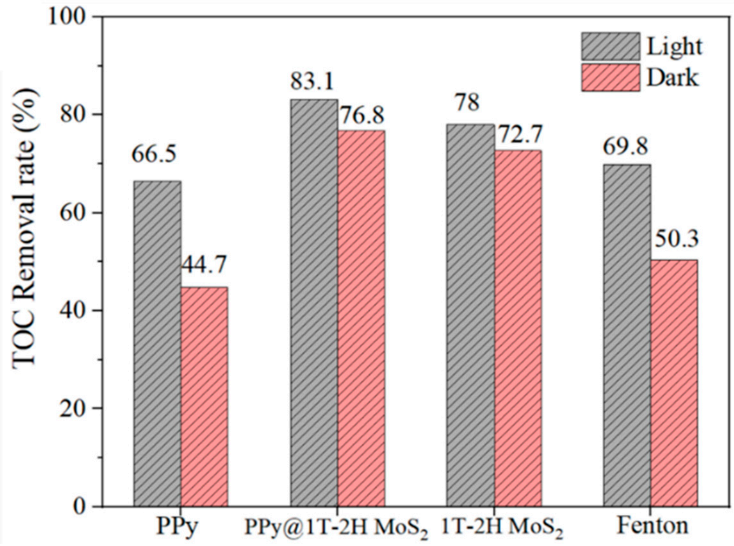

Figure S5 TOC analysis of MB in photocatalytic degradation process by the traditional Fenton method, pure PPy, 1T-2H MoS_2, and PPy@1T-2H MoS_2 catalysts. The experimental conditions consisted of 50 mg of catalyst, 5 mg of $FeSO_4$, and 500 μL of H_2O_2 in 200 mL of contaminated water ([MB] = 25 mg/L).

Figure S6

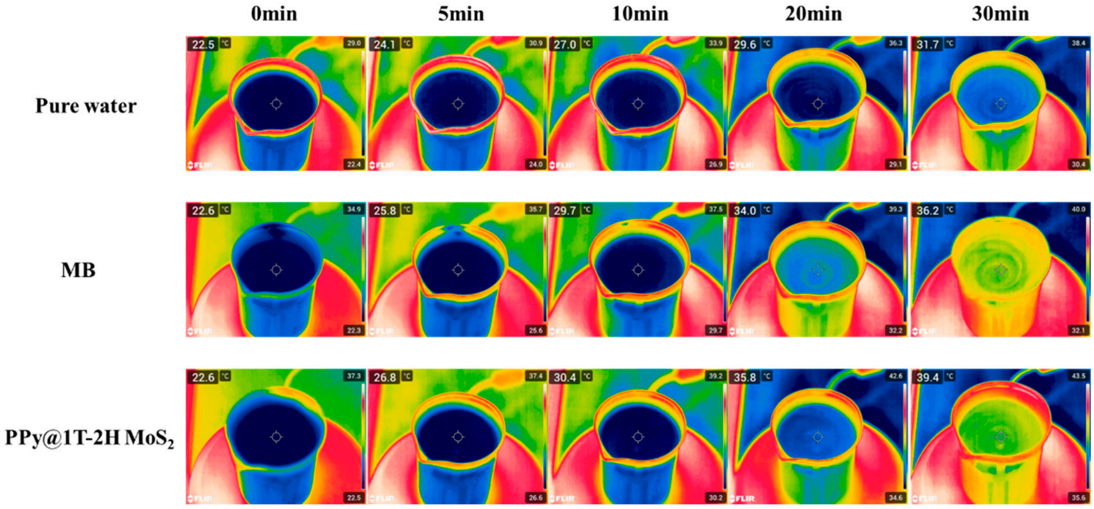

Figure S6 IR images of the temperature change of the upper surface of Water, MB and PPy@1T-2H MoS_2+MB system in 30 minutes.

Figure S7

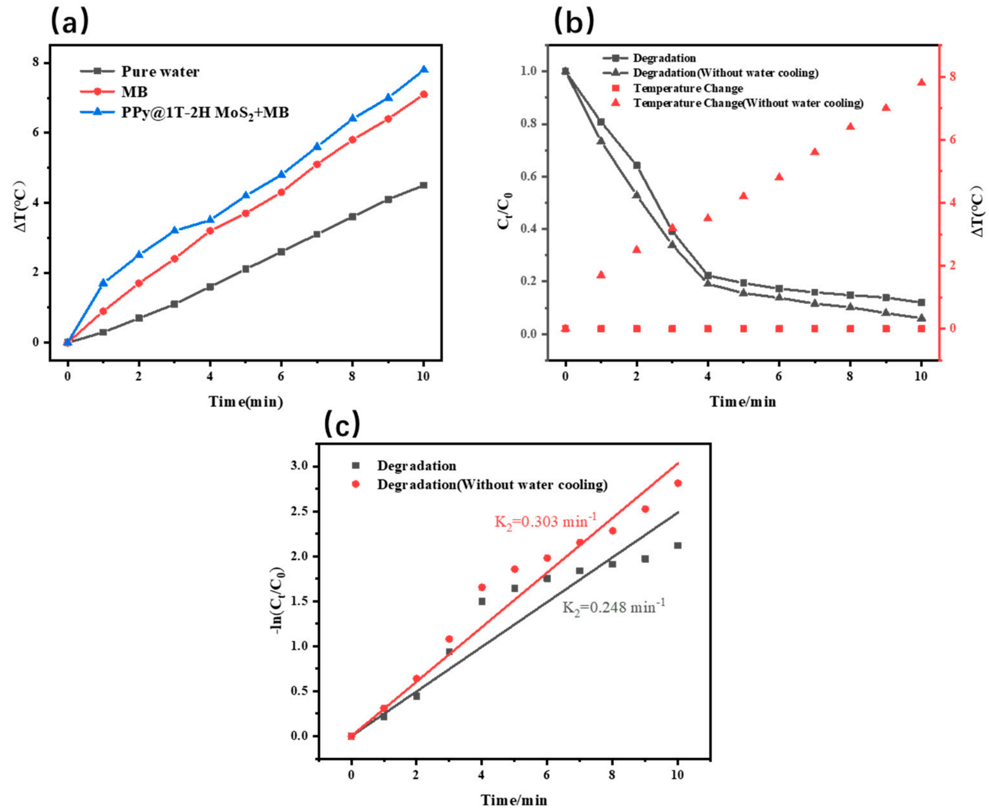

Figure S7 (a) Photothermal heating curves of the pure water, MB-containing water, and PPy@1T-2H MoS_2+MB containing water. (b) Relative concentration changes (C_t/C_0) of MB and Photothermal heating curves of the PPy@1T-2H MoS_2 with and without temperature control. (c) The kinetic fit for MB degradation by PPy@1T-2H MoS_2 composites at various temperatures.

Figure S8

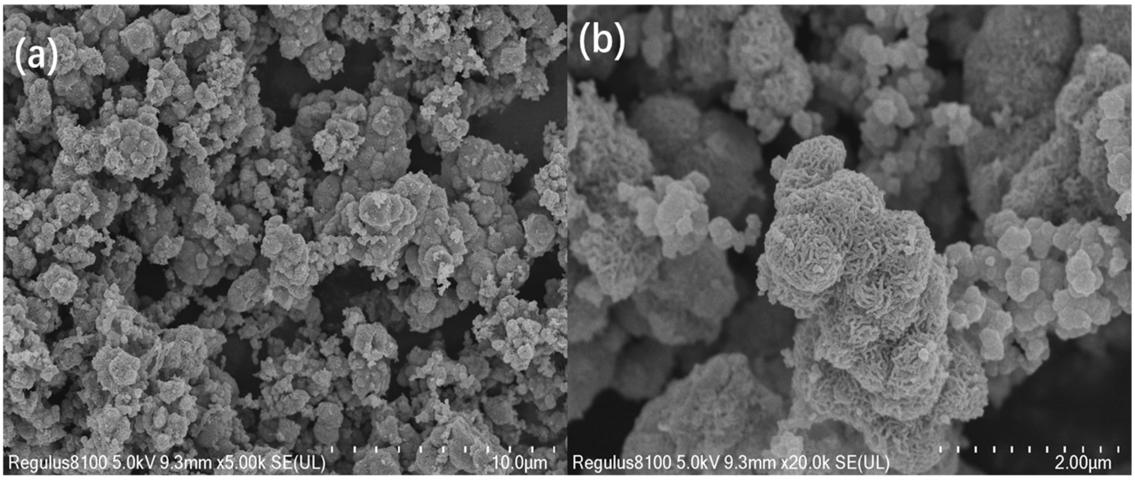

Figure S8 The SEM morphology of the PPy@1T-2H MoS2 after the 5th cyclic test.

Figure S8 shows the morphology of the PPy@1T-2H MoS_2 after the 5th cyclic testament. A spherical morphology was observed. And the composites are consisted of aggregated PPy coated nanosheets. It can be observed that the cyclic measurement did not alter the morphology of PPy@1T-2H MoS_2, indicating the structure stability of the composites.

Figure S9

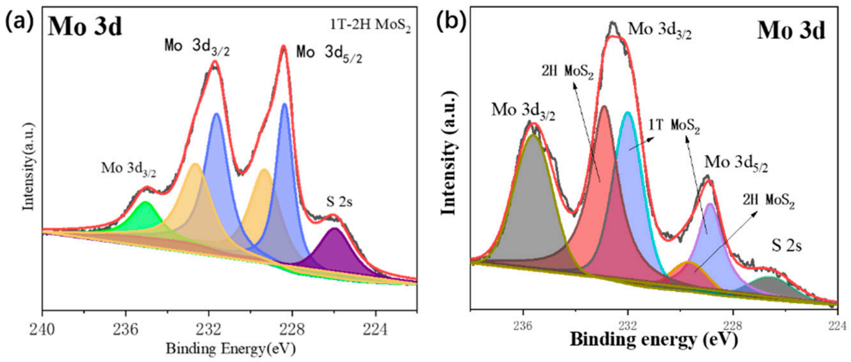

Figure S9. The Mo 3d XPS spectrum of 1T-2H MoS2 (a) before the first test and (b) after the 5th cyclitic test.

The Mo 3d XPS spectrum of 1T-2H MoS2 after 5th cyclitic test of the 1T-2H MoS2 is given in Figure S9(b), in which six peaks were divided in Mo 3d. Among the peaks, the peaks located at 231.9, 228.9, 232.7, and 229.7 eV, corresponding to Mo^{4+}, and 3d3/2 and 3d5/2 in the 1T phase, and Mo^{4+}, 3d3/2 and 3d5/2 in the 2H phase. The peak at 225.3 eV is associated with the S 2s of the unsaturated sulfur atoms in MoS_2, and the peak located at 235.4 eV might be associated with Mo(VI) $3d_{3/2}$, which may be the result of the partial oxidation of MoS_2 to MoO_3. When we compared to the Mo 3d XPS spectrum in Fig. S9, it was observed that the shape of the spectra changed significantly, indicating the changes of the1T-2H MoS2 during cyclic measurement. The intensity of the peak observed at 232.7 eV obvious enhanced, while the decrease of the peak at 231.9 eV, which indicated a decrease in the phase of the 1T component and an increase in the 2H phase. We compared the compositional changes of 1T and 2H phases in MoS_2 samples before the first and after 5 cycles test of the 1T-2H MoS_2 and PPy@1T-2H MoS_2 catalysts by integrating the peaks of the corresponding 2H and 1T phases in the XPS plain. The results were summarized in Table S2.

Table S1

Table S1: Sample elemental composition weight percent ratio.

	1T-2H MoS_2	PPy@1T-2H MoS_2
C	14.8%	27.5%
O	9.7%	5.3%
S	22.9%	17.4%
Mo	52.6%	45.3%
N		4.4%

Table S2

Table S2: Changes in the quantization ratio of 2H to 1T phase in1T-2H MoS_2 during the cyclitic test.

	2H phase		*1Tphase*	
Cyclic number	0 th	5 th	0 th	5 th
1T-2H MoS_2	38.3%	61%	61.7%	39%
PPy@1T-2H MoS_2	38.2%	45.2%	61.8%	54.8%
